# MicroRNA-125b-2 overexpression represses ectodermal differentiation of mouse embryonic stem cells

**DOI:** 10.3892/ijmm.2015.2238

**Published:** 2015-06-08

**Authors:** SHANSHAN DENG, YANLI ZHANG, CHUNDI XU, DUAN MA

**Affiliations:** 1 Key Laboratory of Molecular Medicine, Ministry of Education, Department of Biochemistry and Molecular Biology, Institutes of Biomedical Sciences, Shanghai Medical College, Fudan University, Shanghai 200032, P.R. China; 2 Non-conding RNA and Drug Discovery Laboratory, School of Medical Laboratory Science, Chengdu Medical College, Chengdu, Sichuan 610500, P.R. China

**Keywords:** microRNA, miR-125b-2, mouse embryonic stem cells, embryo bodies, neuron

## Abstract

microRNAs (miRNAs or miRS) have been demonstrated to be essential for neural development. miR-125b-2, presented on human chromosome 21, is overexpressed in neurons of individuals with Down syndrome (DS) with cognitive impairments. It has been reported that miR-125b-2 promotes specific types of neuronal differentiation; however, the function of miR-125b-2 in the early development of the embryo has remained to be fully elucidated. In the present study, a mouse embryonic stem cell (mESC) line was stably transfected with a miR-125b-2 lentiviral expression vector and found that miR-125b-2 overexpression did not affect the self-renewal or proliferation of mESCs. However, miR-125b-2 overexpression inhibited the differentiation of mESCs into endoderm and ectoderm. Finally, miR-125b-2 overexpression was found to impair all-*trans*-retinoic acid-induced neuron development in embryoid bodies. The findings of the present study implied that miR-125b-2 overexpression suppressed the differentiation of mESCs into neurons, which highlights that miR-125b-2 is important in the regulation of ESC differentiation. The present study provided a basis for the further identification of novel targets of miR-125b-2, which may contribute to an enhanced understanding of the molecular mechanisms of ESC differentiation.

## Introduction

MicroRNAs (miRNAs/miRs) are small, non-coding RNAs. They were first identified as developmental mediators in *Caenorhabditis* (*C.*) *elegans* (1,2). It is well known that miRNAs have critical roles in the regulation of gene expression. They are initially transcribed as long RNAs and are then processed by two RNase complexes, Drosha and Dicer, into 22-nucleotide duplexes. These duplexes are loaded into RNA-induced silencing complexes (3,4). The effect of miRNA-mediated modulation of gene expression has been documented across the animal kingdom during numerous steps of neuronal development, from early neurogenesis to synaptogenesis (5–8). Abundant and diverse miRNA expression has also been reported in the central nervous system (9–11).

The presence of three copies of all or part of human chromosome 21 (Hsa21) results in the constellation of physiological traits known as the Down syndrome (DS), also called trisomy 21 (12). Bioinformatic analysis has demonstrated that Hsa21 harbors five miRNA genes, miR-99a, let-7c, miR-125b-2, miR-155 and miR-802 (12,13). miRNA expression profiling, miRNA reverse transcription-quantitative polymerase chain reaction (RT-qPCR), and miRNA *in situ* hybridization experiments have shown that the expression of these miRNAs is markedly higher in fetal brain and heart specimens from individuals with DS than that in samples from age- and gender-matched controls (12,14,15). miR-125b, a homolog of lin-4, was first discovered in *C. elegans*, in which it regulates developmental timing (1). Ectopic expression of miR-125b can increase the relative number of differentiated SH-SY5Y cells that show neurite outgrowth (16). miR-125b is upregulated during the differentiation of human neural progenitor ReNcell VM cells, and high levels of miR-125b have been shown to promote neurite outgrowth in these cells (16). miR-125b also affects dendritic spine morphology. NR2A, which is a subunit of NMDA receptors and affects synaptic plasticity, is a target of miR-125b (17). In hippocampal neurons, NR2A expression is negatively regulated through its 3′-untranslated region by fragile X mental retardation 1, miR-125b and argonaute 1 (17). In poorly differentiated cerebellar granule cell progenitors (GCPs), miR-125b-2 is downregulated, but it promotes GCP differentiation and antagonizes the effects induced by sonic hedgehog (Shh) via targeting activating components of the Hh signaling pathway (18). The present study was performed to assess the association of miR-125b with the nervous system.

Two recent studies have demonstrated the contribution of miR-125b to early neuronal development in embryos (19,20). These studies used the mouse embryonic stem cell (mESC) lines R1 mESCs or E14Tg2a as a model to demonstrate that miR-125b is associated with a specific step during neural differentiation of mESCs. Ectopical expression of miR-125b did not affect the self-renewal of undifferentiated ESCs. However, the expression of a number of miRNAs changed significantly during ESC differentiation, among which miR-125b showed a marked reduction as compared with that in the control. Another study from 2012 showed that overexpression of miR-125b did not affect the ectoderm and neuron differentiation in mESCs (19), which was in contrast with a study from 2013, which reported that the ectopic expression of miR-125b blocked ESC differentiation at the epiblast stage (20). Furthermore, exploration of the targets of miR-125b led to the discovery of two distinct targets, Lin28 and Dies1 (19,20).

The present study investigated the cellular function of the overexpression of miR-125b-2 in mESCs. Stable miR-125b-2-expressing mESC lines were established, and it was shown that the ectopic expression of miR-125b-2 did not affect the self-renewal and proliferation of mESCs. To elucidate the underlying mechanism and the function of miR-125b-2 in the neuronal differentiation of ESCs, ESC-specific germ layer markers characteristic for endoderm, ectoderm and mesoderm were assessed in embryoid bodies. The findings of the present study highlighted an important role of miR-125b-2 in the regulation of ESC germ layer differentiation and revealed a novel mechanism for cell lineage determination and neuronal differentiation.

## Materials and methods

### Cell culture

The mouse ESC line (mESC), E14Tg2a (American Type Culture Collection, Manassas, VA, USA), was kindly provided by Professor Ping Li (Key Laboratory of Molecular Medicine, Fudan University, Shanghai, China). Cells were maintained on feeder-free, gelatin-coated plates (Gibco-BRL, Invitrogen Life Technologies, Carlsbad, CA, USA) in the following medium: Dulbecco’s modified Eagle’s medium (DMEM; Thermo Fisher Scientific, Waltham, MA, USA) supplemented with 2 mM glutamine, 100 U/ml penicillin/streptomycin, 1 mM sodium pyruvate (all from Invitrogen Life Technologies), 1 mM non-essential amino acids (Invitrogen Life Technologies), 0.1 mM l-mercaptoethanol (Sigma-Aldrich, St. Louis, MO, USA), 15% fetal bovine serum (FBS; Thermo Fisher Scientific), and 10^3^ U/ml leukemia inhibitory factor (LIF; Millipore, Billerica, MA, USA). The 293T cells were obtained from Professor Ping Li were cultured in high-glucose DMEM supplemented with 10% FBS at 37°C, with 5% CO_2_ for maintenance.

### Plasmid constructs, viral packaging and ESC transfection

Mouse genomic DNA was purified from the mESC line, E14Tg2a, using GenElute™ Mammalian Genomic DNA Miniprep Kits (Sigma-Aldrich, St. Louis, MO, USA) according to the manufacturer’s insrtuctions. The coding regions of mouse miR-125b-2 were amplified by polymerase chain reaction (PCR) of mouse genomic DNA. [PCR reactions were performed in a total volume of 50 *µ*l consisting of 1 *µ*l of mouse genomic DNA, 10 *µ*l of 5X Prime STAR™ Buffer, 4 *µ*l dNTP Mixture (2.5 mM), 1 *µ*l of each primer (10 *µ*M), and 0.5 *µ*l Prime STAR™ HS DNA Polymerase (2.5 U/*µ*l) (Takara Bio, Inc., Dalian, China). PCR amplifications were carried out on a ThermoHybaid PCR express (Thermo Fisher Scientific, Waltham, MA, USA) and PCR products were analyzed by electrophoresis on a 2.0% agarose gel (Biowest, Spain) containing 0.5 *µ*g/ml of ethidium bromide. Gel images were captured and analyzed using the Quantity One System (Bio-Rad, Hercules, USA)]. They were inserted into the *Age*I and *Eco*RI sites of the pLKO.1 vector. pLKO.1-miR-125b-2 lentiviral vectors combined with packaging plasmids, pMD2.G and psPAX2, were co-transfected into 293T cells using Lipofectamine 2000 reagent (Invitrogen Life Technologies) according to the manufacturer’s instructions. All plasmids, such as, pLKO.1, pMD2.G and psPAX2 were kindly provided by Professor Ping Li (Key Laboratory of Molecular Medicine, Fudan University). Virus-containing supernatant was collected 48 h after transfection and filtered through 0.45-*µ*m filters (Millipore). ESCs were incubated in the virus supernatant supplemented with 4 mg/ml polybrene (Sigma-Aldrich, St. Louis, MO, USA) for 48 h and then the cells were re-plated in fresh mESC culturing medium. Puromycine (Sigma-Aldrich, St. Louis, MO, USA) was added at a final concentration of 2 mg/ml and resistant colonies were selected after 1 week. Pure lentivirus served as a negative control.

### Cell proliferation assays

Cell proliferation was evaluated using the Cell Counting Kit-8 (CCK-8; Dojindo Laboratories, Kumamoto, Japan) according to manufacturer’s instructions. Cells at 12 h post-transfection were seeded into 96-well plates at 5,000 cells/well. Following 24, 48, 72, 96 and 120 h of transfection, 10 *µ*l CCK-8 solution was added to each well. The plate was incubated for 1–4 h in a humidified CO_2_ incubator at 37°C and the absorbance was measured at 450 nm using a Model 680 microplate reader (Bio-Rad Laboratories, Hercules, CA, USA).

### Embryoid body culture

mESC differentiation was induced by transferring ~1,000 cells in 15 *µ*l differentiation medium onto the lid of a 100-mm dish. The cells were cultured for nine days as a hanging drop to facilitate the formation of embryoid bodies (EBs). Each dish contained ~80 embryoid bodies. These were cultured in differentiation medium containing DMEM supplemented with 2 mM glutamine, 100 U/ml penicillin/streptomycin, 1 mM sodium pyruvate, 1 mM non-essential amino acids, 0.1 mM l-mercaptoethanol and 15% FBS. EB medium was changed every other day.

### Neuronal differentiation

The mESCs were grown for four days to form unattached EBs in differentiation medium containing DMEM supplemented with 2 mM glutamine, 100 U/ml penicillin/streptomycin, 1 mM sodium pyruvate, 1 mM nonessential amino acids, 0.1 mM L-mercaptoethanol and 15% FBS. After 4 days of embryoid body formation the cells were treated with 1 *µ*M all-*trans*-retinoic acid (Sigma-Aldrich, Buchs, Switzerland) for an additional 4 days. These EBs were digested and transferred to poly-d-lysine/laminin-coated tissue culture dishes (Sigma-Aldrich, St. Louis, MO, USA). The cells were then incubated in DMEM with 10% heat-inactivated FBS to induce neuronal differentiation.

### RNA extraction and reverse transcription quantitative PCR (RT-qPCR)

Total RNA from mESCs was isolated using the TRIzol reagent (Invitrogen Life Technologies) according to the manufacturer’s instructions, and the concentration was determined by the ratio of the absorbance at 260 to that at 280 nm using a NanoDrop^®^ ND-1000 spectrophotometer (Thermo Fisher Scientific). To measure the content of miR-125b-2, 500 ng total RNA was poly-A tailed and reverse transcribed to cDNA using an All-in-One™ miRNA qRT-PCR Detection kit (Cat. no. AOMD-Q050; GeneCopoeia Inc., Rockville, MD, USA) according to the manufacturer’s instructions. Real-time PCR was then performed using an ABI7300 Real-Time PCR System (Applied Biosystems, Foster City, CA, USA) with miRNA-specific forward and reverse primers. Each reaction was performed with 2 *µ*l template cDNA, 10 *µ*l 2X All-in-One qPCRMix, 2 *µ*l of each primer (2 *µ*M), 0.4 *µ*l 50X ROX Reference Dye, and water to adjust to a final volume of 20 *µ*l. All reactions were incubated on a 96-well plate at 95°C for 10 min, followed by 40 cycles of 95°C for 10 sec, 65°C for 20 sec and 72°C for 10 sec. Statistical analysis was performed using SDS software version 1.4.1 (Applied Biosystems). For the analyses of marker genes of mESCs, EBs and neurons, RNA (1 *µ*g) of each sample was used for reverse transcription with the Prime Script^®^ RT reagent kit (Takara Bio, Inc.) using Oligo(dT) Primer at 37°C for 15 min, followed by 85°C for 5 sec. The amplified cDNA was quantified using SYBR^®^ Premix Ex Taq™ (DRR041A; Takara Bio, Inc.) according to the manufacturer’s instructions. Each reaction was performed with 2 *µ*l template cDNA, 10 *µ*l 2X SYBR Premix Ex Taq. 0.4 *µ*l of each primer (10 *µ*M), 0.4 *µ*l 50X ROX Reference Dye, and water to adjust to a final volume of 20 *µ*l. All reactions were incubated on a 96-well plate at 95°C for 30 sec, followed by 40 cycles of 95°C for 5 sec and 60°C for 31 sec. Real-time PCR was then performed using the same qPCR apparatus and statistical analysis was performed using the same software. The resulting cDNA was then amplified by qPCR using the primers listed in Table I. The housekeeping genes U6 and the GAPDH were used to normalize the samples, using the 2^−∆∆Ct^ method. All primers were obtained from GeneCopoeia.

### Immunofluorescence analysis

Cells were permeabilized with 0.25% Triton X-100 (Sigma-Aldrich, St. Louis, MO, USA) for 10 min at 37°C and then fixed with 4% paraformaldehyde (Sigma-Aldrich, St. Louis, MO, USA) in phosphate-buffered saline (PBS) for 15 min at room temperature. The fixed cells were blocked for 20 min with PBS containing 5% bovine serum albumin (BSA; Sigma-Aldrich, St. Louis, MO, USA). Next, the cells were incubated for 16 h at 4°C with mixtures containing primary antibodies specific to the ESC markers, Nanog (1:100; species raised in: rabbit; specificity: rat, human and mouse; monoclonal antibody; Millipore, AB5731), octamer-binding transcription factor 4 (Oct4; 1:100; species raised in: mouse; specificity: human and mouse; monoclonal antibody; Millipore, MABD76) and sex-determining region Y-box 2 (Sox2; 1:100; species raised in: mouse; specificity: human and mouse; monoclonal antibody; Millipore, MAB4343) in PBS containing 1% BSA. The cells were washed three times with PBS containing 1% BSA. As the secondary antibody, goat anti-mouse immunoglobulin G conjugated to fluorescein isothiocycanate (Sigma-Aldrich, St. Louis, MO, USA) was applied at dilutions of 1:500. The Hoechst 33342 reagent (Sigma-Aldrich, St. Louis, MO, USA) was used to detect the nuclei in cells. After washing with PBS for three times, the cells were analyzed using a confocal scanning laser fluorescence microscope (Model FV300; Olympus, Tokyo, Japan).

### Statistical analysis

Values are expressed as the mean ± standard error of three independent experiments. Statistical significance of differences was calculated using Prism software (version 4.0a; GraphPad Software, Inc., La Jolla, CA, USA) by one-way analysis of variance. P<0.05 was considered to indicate a statistically significant difference between values.

## Results

### miR-125b-2 overexpression does not affect the pluripotency and self-renewal of mESCs

**Figure 1:**
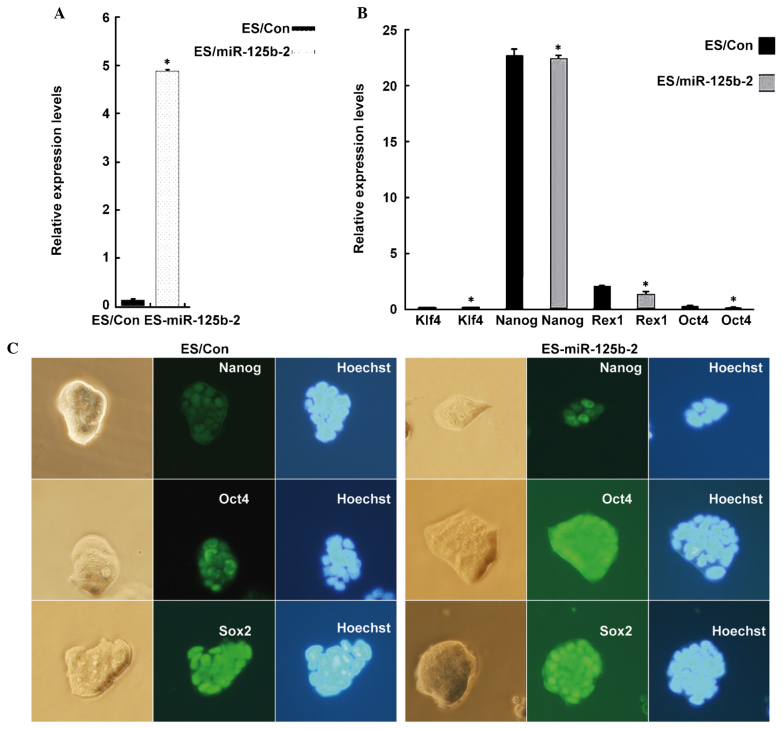
miR-125b-2 does not affect the pluripotency of ESCs. (A) RT-qPCR analysis of miR-125b-2 expression in ESCs transfected with empty vector and miR-125b-2. U6 was used as loading control. (B) RT-qPCR analysis of self-renewal marker gene expression. GAPDH was used as loading control. Groups: ES/Con, empty vector-transfected ESCs; ES/miR-125b-2, miR-125b-2-transfected ESCs. Values are expressed as the mean ± standard error of data from one representative of three experiments, performed in triplicate. ^*^P<0.05 compared with the ES/Con group. (C) Phase contrast microscopy images of miR-125b-2-transfected (right) and control (left) cells. Immunofluorescence staining for the stem cell pluripotency markers Nanog, Oct4 and Sox2. Cell nuclei were stained by Hoechst. RT-qPCR, reverse transcription quantitative polymerase chain reaction; ESCs, embryonic stem cells; miR, microRNA; Sox2, sex-determining region Y-box 2; Oct4, octamer-binding transcription factor 4; KLF4, kruppel-like factor 4.

To determine the function of miR-125b-2 in the maintenance of pluripotency and self-renewal, ESCs that overexpressed miR-125b-2 were established by transfection with a pLKO.1 lentiviral expression vector. According to addgene (http://www.addgene.org), the plasmid psPAX2 produces a higher titer than pCMV-dR8.2 dvpr and contains a robust CAG promoter for efficient expression of packaging proteins (21–23). Therefore, a lentiviral pLKO.1 vector containing an miR-125b-2 expression vector, a psPAX2 packaging vector and a pMD2.G envelope vector were combined at a ratio of 4:3:1. Twenty-four hours after transfection, the cells were selected using puromycin. After one week, the overexpression of miR-125b-2 was verified by RT-qPCR. The results showed that the expression levels of miR-125b-2 in mESCs were 36 times higher than those in the controls (Fig. 1A), which indicated the successful establishment of stably miR-125b-2-expressing mESCs. Next, to determine the effects of miR-125b-2 on the self-renewal of mESCs, the expression of the mESC markers Klf4, Nanog, Rex1 and Oct4 was detected by RT-qPCR. There were no significant differences in the expression levels of self-renewal markers between miR-125b-2-transfected cells and control cells (Fig. 1B) Furthermore, Nanog, Sox2 and Oct4 were detected by immunohistochemistry. Fluorescence microscopy showed that all four markers remained detectable in miR-125b-2-transfected cells. Phase-contrast images of the morphology of the colonies in the presence of LIF showed no obvious differences in morphology between miR-125b-2-transfected cells and control cells (Fig. 1C). These results indicated that the overexpression of miR-125b-2 had no influence on the maintenance of mESCs.

### miR-125b-2 overexpression does not promote ESC proliferation

**Figure 2:**
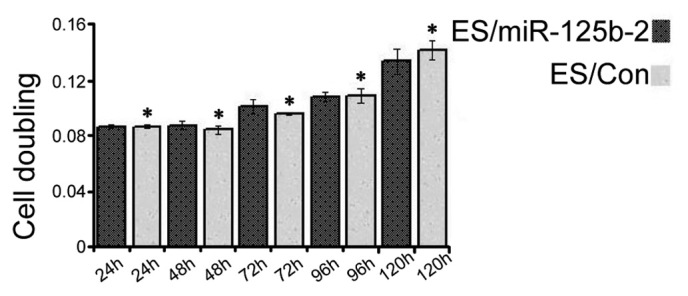
Cell counting kit-8 analysis of the growth of ES/Con and ES/miR-125b-2 cells. No significant difference in ESC proliferation was observed between the groups at all time-points. ES/Con, empty vector-transfected ES cells; ES/miR-125b-2, miR-125b-2-transfected ESCs. Values are expressed as the mean ± standard error of data from one representative of three experiments, performed in triplicate. ^*^P<0.05 compared to ES/Con. ESCs, embryonic stem cells; miRNA, microRNA.

miRNAs have important roles in living organisms and regulate stem cell proliferation (24). To investigate the biological effects of miR-125b-2 on ESC proliferation, CCK-8 was added at various time-points (24, 48, 72, 96 and 120 h) after transfection (25,26). Overexpression of miR-125b-2 did not significantly stimulate the growth of mESCs as compared with that in the controls (P<0.05) (Fig. 2), which implied that miR-125b-2-overexpression had no distinct effect on ESC proliferation.

### miR-125b-2 overexpression inhibits the differentiation of mESCs into endoderm and ectoderm, but not mesoderm

**Figure 3:**
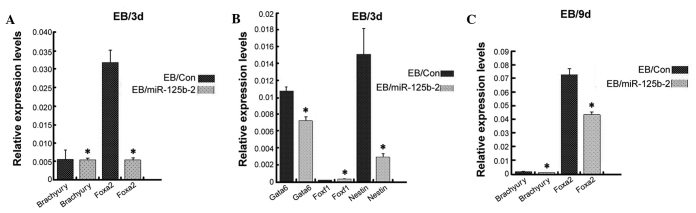
Analysis of gene expression profiles of EB/Con and EB/miR-125b-2 groups at various time-points. (A) The expression levels of endodermal marker Foxa2 and mesoderm marker Brachyury were detected in day-3 EBs. (B) The expression levels of endodermal marker Gata6, mesodermal marker Foxf1 and the parietal ectoderm marker Nestin were detected in day-3 EBs. (C) The expression levels of endodermal marker Foxa2 and mesoderm marker Brachyury were detected in day-9 EBs. The individual values were normalized against the endogenous standard gene GAPDH. Groups: EB/Con, empty vector-transfected EBs; EB/miR-125b-2, miR-125b-2-transfected EBs. Values are expressed as the mean ± standard error of data from one representative of three experiments, performed in triplicate. ^*^P<0.05 compared to ES/Con. EBs, embryoid bodies; Fox, forkhead box; miR, microRNA.

The self-renewal capacity and differentiation potential are hallmarks of stem cells (24). In the past few years, miR-125b was shown to be an important factor involved in stem cell development by regulating the differentiation of stem cells (19,20,27). To evaluate the effect of miR-125b-2 on the direction of ESC differentiation, transfected and control ESCs were cultured in suspension for four days to form EBs (28). RT-qPCR analysis was performed to detect markers of endoderm, ectoderm and mesoderm on day-3 and day-9 EB cells. The levels of Foxa2 and Gata6 (29,30), which are expressed by all extra-embryonic endodermal cells, were significantly decreased in miR-125b-2-overexpressing EBs compared with those in the control EBs (P<0.05) (Fig. 3). The ectoderm marker Nestin (30,31) was also significantly decreased in miR-125b-2 transfectants compared with that in the control EBs (P<0.05) (Fig. 3B). However, there were no differences in the expression levels of the mesoderm markers Brachyury and Foxf1 (32) (Fig. 3). These results suggested that miR-125b-2 overexpression suppressed the differentiation of mESCs into endoderm and ectoderm, while there was no obvious influence on the mesodermal differentiation of ESCs.

### miR-125b-2 overexpression reduces neural progenitor differentiation

**Figure 4:**
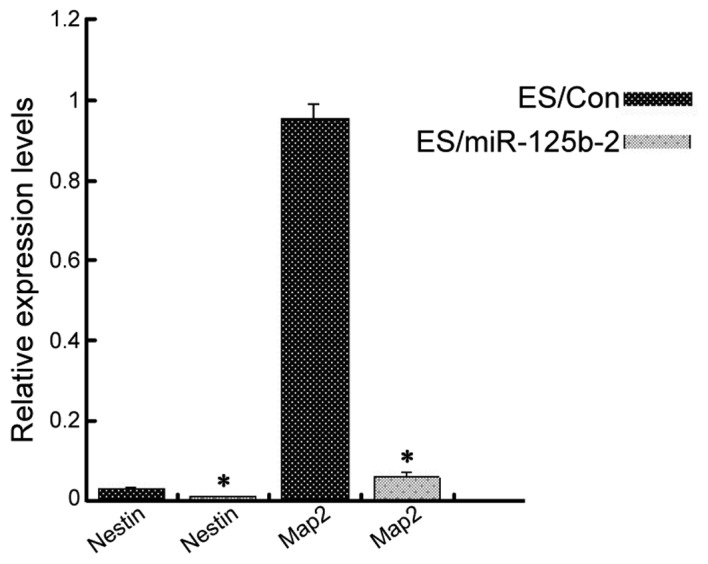
Analysis of neuronal marker profiles in ES/Con and ES/miR-125b-2 groups. The expression levels of neuronal markers Nestin and Map2 were detected by reverse transcription quantitative polymerase chain reaction. The individual values were normalized against the endogenous standard gene GAPDH. Values are expressed as the mean ± standard error of data from one representative of three experiments, performed in triplicate. ^*^P<0.05 compared to ES/Con. ES/Con, empty vector-transfected ESCs; ES/miR-125b-2, miR-125b-2-transfected ESCs; ESC, embryonic stem cell; miR, microRNA; Map, microtubule-associated protein.

Ectoderm is one of the three classic germ layers in the early mouse embryo, with the capacity to develop into the central nervous system (33). In order to determine the impact of miR-125b-2 overexpression on the nervous system, RA was used to induce EB differentiation into neuronal cells (34). ESCs were first cultured in suspension for four days to form EBs. RA was then added to the medium and the cells were incubated for another four days. The cells were then adherently cultured for 3–6 days. The neuron-specific markers Nestin and Map2 were analyzed using RT-qPCR (35). There was a significant decrease in the expression of the two neuronal markers in the ES/miR-125b-2 group as compared with those in the control group (Fig. 4). These results suggested that downregulation of miR-125b-2 may be required to induce the differentiation of ESCs.

## Discussion

The present study showed that miR-125b-2 has an important role in mouse embryonic stem cells (mESCs) by inhibiting the differentiation of mESCs into endoderm and ectoderm without affecting their proliferation, mesodermal differentiation and self-renewal. Functional genetic studies on EBs treated with RA further indicated that miR-125b-2 overexpression impaired neuron development. The present study demonstrated that it is necessary to further investigate the regulatory mechanisms of the effects of miR-125b on the self-renewal and differentiation of ESCs.

A stably miR-125b-2-overexpressing mESC line E14Tg2A was established by transfection with an miR-125b-2 expression lentivirus. RT-qPCR analysis confirmed that miR-125b-2 was expressed in undifferentiated mESCs (Fig. 1A); however, it has remained impossible to identify the expression levels without the threshold. For example, Tarantino *et al* (36) reported that miR-125b is undetectable in undifferentiated cells and is induced upon differentiation in the two mESC lines E14Tg2A and MPI; however, it was not detectable in R1 mESCs by microRNA array. By contrast, Wang *et al* (19) reported that miR-125b expression was detected in R1 mESCs using microRNA array screening. They further showed that miR-125b is highly enriched in undifferentiated mESCs as compared with other expressed miRNAs, while it is markedly downregulated during early ESC differentiation (19). Solozobova and Blattner (37) have shown that expression of miRNA-125b-2 during the process of EB formation is significantly lower in all mESC lines (R1, D3 and CGR8) than that in differentiated cells. The expression of miR-125b-2 in brains of children with DS was found to be 1.5 times higher than that in normal brains (14). In agreement with these data, miR-125b-2 was shown to be highly expressed in numerous adult mouse tissue types. The discrepancies among the abovementioned previous studies may be due to the different ESC lines and differentiation protocols used. The present study used RA to induce differentiation, as its effects are similar to natural early embryonic development (38). The differentiation protocol used in the present study induces slow and more physiological differentiation which mainly affected neurons (14).

The results of the present study demonstrated that miR-125b is essential for the proper differentiation of ESCs, which is consistent with the results recently observed in mESCs (19,20). In the present study, the expression of four ESC self-renewal markers was found to be similar among miR-125b-2-overexpressing and control cells, and no major change in morphology was observed among them. Of note, a proportion of miR-125b-overexpressing cells were resistant to differentiation into endoderm and ectoderm. This regulatory role of miR-125b was confirmed by the observations of previous studies, which reported that the downregulation of miR-125b is required for the initiation of ESC differentiation (19,20). In addition, it is known that miRNAs have important roles in cell cycle regulation of ESCs (39). Compared with somatic cells, ESCs are characterized by a cell cycle with a shortened G1 phase as an adaptation to the rapid growth during early embryonic development. Furthermore, the present study tested the proliferation of miR-125b-2-overexpressing mESCs using the CCK-8 assay. miR-125b-2 was found to have no significant effect on the proliferation of mESCs. A previous screening-based study, which examined the effect of 461 individually re-introduced miRNAs on the proliferation of DGCR8-null cells showed that the defective proliferation was rescued by 14 different miRNAs, including miR-290, miR-302 and the miR-17-92 cluster (40 and refs therein). These findings showed that miR-125b may not be an ESC-specific cell cycle-regulating miRNA. Therefore, these observations, together with the findings of the present study, suggested a distinctive role of miR-125b in the lineage commitment of ESCs as well as tissue/organ generation.

The results of the present study further showed that miR-125b is acts as a regulator of ESC-specific germ layer commitment. Wang *et al* (19) reported that ectopically expressed miR-125b-2 can impair the expression of endoderm marker genes, which is consistent with these results. It has been demonstrated that the endoderm forms the respiratory and digestive tracts, which has implications for diseases of the endoderm, including cystic fibrosis and cancer (41). In contrast to the marked inhibitory role of miR-125b on endodermal and ectodermal differentiation, the present study has shown that overexpression of miR-125b-2 did not affect the expression of mesoderm-associated markers and therefore the mesodermal differentiation of ESCs. Furthermore, the ectoderm forms the central nervous system (41); therefore the decreased RA-induced differentiation of ESCs into neurons following overexpression of miR-125b-2, as indicated by reduced levels of neuroectodermal markers, was in line with the decreases in ectodermal differentiation. However, other studies have shown that miR-125b-2 promote neuronal differentiation of SH-SY5Y, GCP and P19 cells as well as hippocampal neurons (16–18). Boissart *et al* (42) showed that miR-125b-2 potentiated early neuronal specification of human embryonic stem cells (hESCs). hESC neurons were induced by N2B27 medium supplemented with fibroblast growth factor 2. Wu and Belasco (43) produced similar results using a specific non-mESC line, mouse P19 embryonal carcinoma cells. Differences in experimental protocols may in part explain the differences between the results of the present study and those of previous studies. The discrepant conclusions may also be explained by the limitations of the methods of the present study regarding mESC differentiation *in vitro*. Thus, it cannot be excluded that miR-125b also regulates ectoderm formation and neural differentiation, which therefore requires further study.

Of note, miR-125b was shown to target Lin28 in cardiac differentiation, whereas it targets Dies1 in neuronal differentiation (20). A bioinformatics study and a luciferase reporter assay have been performed in our group and will be published shortly. The preliminary results showed that miR-125b inhibited the expression of at least five genes, among which at least two genes were associated with nervous system development (data not shown). In addition, miR-125a was shown to be essential for the proper differentiation of ESCs (44), which suggested that at least two miRNAs work cooperatively to inhibit Dies1. It is well known that redundancy characterizes miRNA functions: In most cases, one single miRNA targets a number of mRNAs and, on the other hand, one mRNA is often targeted by a number of miRNAs (45). Whether miR-125b coordinately targets Dies1 and other genes requires further investigation.

In conclusion, the results of the present study indirectly demonstrated that miR-125b is required for the initiation of ESC differentiation. It negatively regulates endodermal and ectodermal differentiation and terminal differentiation of neurons, while not affecting mesodermal differentiation. Therefore, the ongoing identification of novel targets of miR-125b will further elucidate the molecular mechanisms of ESC differentiation and may provide tools to direct ESC differentiation toward specific lineages.

## Figures and Tables

**Table I:** Primers used for reverse transcription quantitative polymerase chain reaction.

Name	Forward primer (5′→3′)	Reverse primer (5′→3′)
Oct4	CTGAGGGCCAGGCAGGAGCACGAG	CTGTAGGGAGGGCTTCGGGCACTT
Nanog	AGGGTCTGCTACTGAGATGCTCTG	CAACCACTGGTTTTTCTGCCACCG
Klf4	CAAGTCCCCTCTCTCCATTATCAAGAG	CCACTACGTGGGATTTAAAAGTGCCTC
Rex1	AAGCCGTATCAGTGCACGTTCGAAGGCT	ATGCGTGTATCCCCAGTGCCTCTGTCAT
Gata6	TTGCTCCGGTAACAGCAGTG	GTGGTCGCTTGTGTAGAAGGA
Foxa2	CCCTACGCCAACATGAACTCG	GTTCTGCCGGTAGAAAGGGA
Nestin	CTGAGAACTCTCGCTTGCAGACA	GGAAATGCAGCTTCAGCTTGG
Foxf1	CGGAGAAGCCGCCCTACT	GCGCGCCTGAGAAACTG
Brachyury	GCGGGAAAGAGCCTGCAGTA	TTCCCCGTTCACGTACTTCC
Map2	CATCGCCAGCCTCGGAACAAACAG	TGCGCAAATGGAACTGGAGGCAAC
U6	CTCGCTTCGGCAGCACA	CTCGCTTCGGCAGCACA
GAPDH	GTATGACTCCACTCACGGCAAA	TTCCCATTCTCGGCCTTG
